# An IoT-Enabled Modular 3D Bioreactor for Vascular Tissue Engineering: Design, Fabrication, and Biological Validation

**DOI:** 10.3390/bioengineering13050589

**Published:** 2026-05-21

**Authors:** Belma Nalbant, Ahmet Ozkurt, Taner Akkan, Tufan Egeli, Thomas Pufe, Zeynep Yuce, Tarkan Unek

**Affiliations:** 1 Department of Anatomy and Cell Biology, Uniklinik RWTH Aachen, 52074 Aachen, Germany; tpufe@ukaachen.de; 2 Department of Electrical and Electronics Engineering, Faculty of Engineering, Dokuz Eylul University, Buca, 35360 Izmir, Turkey; ahmet.ozkurt@deu.edu.tr; 3 Department of Electronics and Automation, Izmir Vocational School, Dokuz Eylul University, Buca, 35390 Izmir, Turkey; taner.akkan@deu.edu.tr; 4 Department of General Surgery, Faculty of Medicine, Dokuz Eylul University, Balcova, 35330 Izmir, Turkey; tufan.egeli@deu.edu.tr (T.E.); tarkan.unek@deu.edu.tr (T.U.); 5 Department of Medical Biology, Faculty of Medicine, Dokuz Eylul University, Balcova, 35330 Izmir, Turkey; zeynep.yuce@deu.edu.tr

**Keywords:** bioreactor, vascular tissue engineering, 3D cell culture, HASMC, agarose hydrogel, confocal microscopy, LDH assay, ImageJ analysis, IoT, dynamic culture

## Abstract

Three-dimensional (3D) bioreactor systems are essential for vascular tissue engineering as they provide controlled environments that better mimic physiological conditions compared to static culture systems. In this study, an IoT-enabled modular rotating 3D bioreactor platform was designed, fabricated using Fused Deposition Modeling (FDM), and biologically validated. The system integrates a Wi-Fi-supported ESP8266 controller and a touchscreen human–machine interface (HMI), enabling real-time monitoring and remote operation. Agarose-chitosan-based tubular hydrogel constructs were seeded with human aortic smooth muscle cells (HASMCs) and cultured under dynamic conditions for 14 days. Biocompatibility was assessed using a lactate dehydrogenase (LDH) assay, while cellular distribution and mitochondrial activity were evaluated by confocal microscopy using DAPI and MitoTracker staining. Fluorescence intensity was further quantified using ImageJ, and 3D surface plots were generated to visualize spatial signal distribution. The results demonstrated sustained cell viability with decreasing cytotoxicity over time. Confocal analysis confirmed a homogeneous distribution of cells within the hydrogel matrix, and quantitative fluorescence analysis showed significantly higher MitoTracker intensity compared to DAPI, indicating increased metabolic activity under dynamic conditions. These findings suggest that the developed bioreactor provides a stable, controllable, and effective platform for vascular tissue engineering applications.

## 1. Introduction

Vascular grafts required for surgical procedures are currently obtained from autologous venous grafts, cryopreserved cadaveric tissues, or commercially available synthetic grafts [1,2]. Autologous venous grafts are widely accepted as the clinical gold standard due to their appropriate biocompatibility and functional performance; however, their applications are limited by donor site morbidity, limited availability, and the necessity for additional surgical intervention [3]. Synthetic vascular grafts, despite being clinically used since the mid-twentieth century, are associated with well-documented disadvantages such as mechanical incompatibility, endothelial insufficiency, fibrosis at anastomosis sites, thrombogenicity, risk of infection, and intimal hyperplasia [2,4]. These persistent clinical challenges have motivated the development of tissue-engineered vascular grafts as a promising alternative strategy [5].

The fabrication of vascular tissue constructs relies on a variety of scaffold materials; each selected according to specific structural and biological requirements. Natural polymers such as collagen, chitosan, agarose, and fibrin offer excellent biocompatibility and cell-interactive properties; however, they often exhibit limited mechanical strength. Synthetic polymers, including poly(caprolactone) (PCL), poly (lactic acid) (PLA), poly(lactic-co-glycolic acid) (PLGA), and polyurethane (PU), provide tunable mechanical properties and controlled degradation rates, though they may lack inherent biological activity. Hybrid scaffolds combining natural and synthetic components have therefore emerged as a promising approach to balance biocompatibility with structural performance.

Several fabrication strategies have been developed for vascular tissue engineering, each offering distinct advantages. Electrospinning produces nanofibrous scaffolds that closely mimic the native extracellular matrix architecture, providing structural support and enabling localized therapeutic release [6]. Additive manufacturing and 3D bioprinting offer unparalleled spatial control to fabricate highly customized, patient-specific anatomical structures and complex vascular networks using biocompatible materials [7]. Molding offers a reliable and cost-effective approach to cast standardized tubular constructs with reproducible geometries [8]. Bioreactor-based systems complement all these fabrication approaches by providing dynamic culture environments that promote tissue maturation under physiologically relevant mechanical stimuli.

In addition to these clinical limitations, researchers are increasingly relying on in vitro vascular models to address specific questions in drug development and preclinical research. Traditional two-dimensional (2D) cell culture systems limit their predictive value in pharmacological tests because they do not adequately mimic the three-dimensional architecture and complex cell–cell interactions specific to native vascular tissues [9,10]. As a result, 3D culture strategies such as organoids, microfluidic platforms, bioprinting technologies, and bioreactor-based systems have garnered increasing interest as more physiologically relevant experimental models [10]. In particular, dynamic 3D culture systems have been shown to significantly improve cell viability, metabolic activity, and tissue organization compared to conventional static approaches [11].

Bioreactor systems play a central role in 3D tissue engineering by providing controlled in vitro environments that support tissue maturation and experimental reproducibility. These systems regulate essential physical and chemical parameters necessary to maintain stable culture conditions, such as temperature, nutrient and oxygen transport, medium renewal, and mass transfer [12,13]. Beyond tissue engineering, bioreactors are widely used in drug research and biopharmaceutical production; here, standardized and controllable environments are critical for obtaining reproducible biological results [14,15]. Among the various bioreactor configurations, rotating wall vessel (RWV) systems are particularly advantageous for vascular applications, as they generate low-shear, low-turbulence hydrodynamic conditions that closely mimic the mechanical microenvironment of native blood vessels [16].

Despite their wide range of applications, the design of bioreactor systems is often highly application-specific and requires practical compromises between biological demands, technical feasibility, controllability, usability, and cost. In the context of vascular tissue engineering research, particular emphasis is placed on precise environmental control, modular system design, and adaptability to different experimental configurations [12,17,18,19]. Recent advances in additive manufacturing, particularly fused deposition modeling (FDM), have enabled rapid prototyping of customizable bioreactor components with complex geometries, facilitating the development of accessible platforms for tissue engineering research [20]. Recent studies have shown that standardized design approaches, user-friendly control systems, and improved experimental reproducibility have become increasingly important features of bioreactor platforms developed for vascular research applications [21,22,23]. Recent advances in vascular bio-fabrication, engineered vessel maturation, and perivascular scaffold technologies further highlight the growing translational potential of customizable tissue-engineering platforms for vascular regeneration applications [24,25,26].

Natural polysaccharide-based hydrogels, including agarose and chitosan, have emerged as attractive scaffold materials for vascular tissue engineering due to their tunable mechanical properties, biocompatibility, and ability to support three-dimensional cell growth [27,28,29]. Their combined use in hybrid formulations allows for optimization of both structural integrity and cell–matrix interactions, making them suitable candidates for tubular vascular construct fabrication [28].

In this study, we present the design, production, and biological validation of a modular 3D bioreactor platform specifically designed for vascular tissue engineering. The proposed system integrates FDM production with an IoT-supported ESP8266 controller (Espressif Systems, Shanghai, China) and a touchscreen interface, providing both local and remote operational management. Beyond the technical architecture, the functional capability of the platform was validated through a 14-day culture process using primary human aortic smooth muscle cells (HASMC) seeded on an agarose-chitosan-based hydrogel mandrel. Technical evaluations confirmed the mechanical reliability and remote connectivity of the system, while biological results demonstrated sustained cell viability and widespread distribution within the 3D constructs. To our knowledge, this is among the first studies to combine FDM-fabricated modular design, IoT-enabled remote control, and agarose-chitosan hydrogel scaffolds within a single rotating bioreactor platform for vascular tissue engineering validation. Therefore, this study provides a cost-effective and versatile technical foundation for the development of vascular models produced by tissue engineering under meticulously controlled conditions.

## 2. Materials and Methods

### 2.1. Bioreactor Design

**Figure 1:**
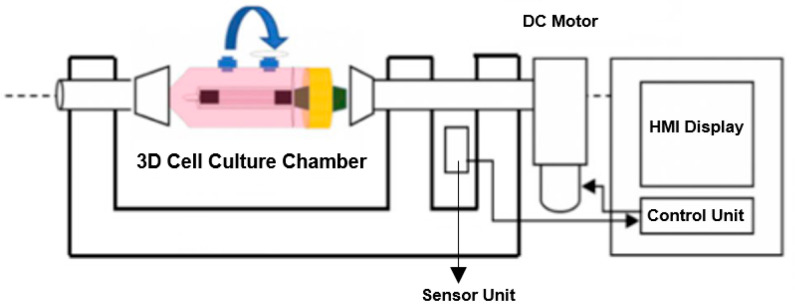
Schematic representation of the rotating bioreactor system and its main components.

The overall architecture of the developed bioreactor system is illustrated in the schematic diagram in Figure 1. The platform comprises three primary functional modules: a rotating chamber for sample accommodation, a motor-driven actuation system for controlled rotation, and a 3.5-inch touchscreen HMI for real-time monitoring and control of rotational speed, duration, and temperature.

**Figure 2:**
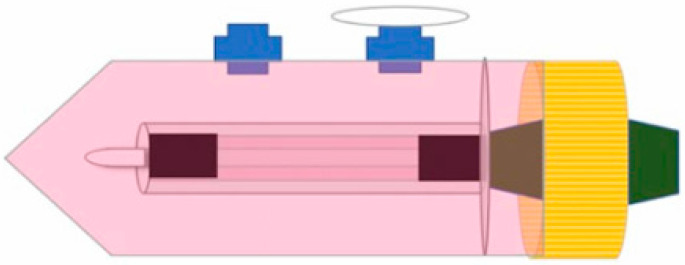
Schematic illustration of the 3D cell culture chamber used for vascular tissue formation. The chamber includes: a gas exchange port equipped with a 0.22 µm sterile filter for continuous CO_2_/O_2_ exchange while maintaining sterility, and a medium exchange port enabling sterile medium replacement without opening the system.

The system’s block diagram was developed using 3D modeling software (123Design; Autodesk, San Francisco, CA, USA) based on a slow-speed actuator configuration. During the design phase, two distinct motor and drive configurations were evaluated to ensure stable mechanical performance. The central mechanical element is the tissue scaffold holder (Figure 2), which integrates a mandrel to support the tubular geometry of the 3D vascular constructs. In our study, the mandrel was designed with a diameter of 4.5 mm and a total length of 100 mm, providing a functional construct-bearing length of 78 mm. To maintain structural stability during cultivation, two gripper stoppers are incorporated to restrict axial movement.

Effective gas exchange is achieved through a specialized lunar-shaped inlet equipped with vessel venting filters (0.22 µm pore size) to ensure sterility. A secondary lunar inlet is dedicated to the periodic replacement of culture medium, allowing for nutrient supply and waste removal without disrupting the system’s internal environment. The detailed medium exchange protocol is described in Section 2.4.

**Figure 3:**
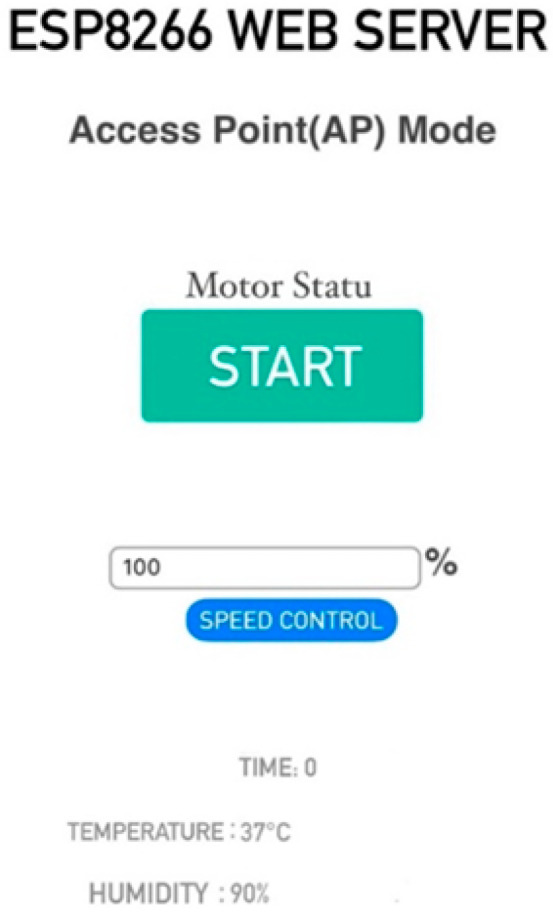
Web-based user interface of the ESP8266-controlled bioreactor system, enabling remote monitoring and adjustment of operational parameters via mobile devices. The interface displays real-time values for rotational speed (%), elapsed operating time, incubator temperature (°C), and relative humidity (%). Remote control functions (start, stop, speed adjustment) are available for use in the event of a system malfunction or when experimental conditions require modification.

User interaction is facilitated through a Nextion touchscreen HMI (ITEAD Studio, Shenzhen, China), enabling precise control over operational parameters. Integrated with an ESP8266-based microcontroller (Espressif Systems, Shanghai, China), the system supports remote management via a web-based interface accessible through mobile devices or tablets (Figure 3). This remote capability is critical for long-term experiments, as it allows parameter adjustment without opening the incubator, thereby minimizing contamination risks. The bioreactor is designed for continuous operation (ranging from hours to months) with three optimized low-speed modes to accommodate various cell growth requirements. The parameters that can be monitored include rotation speed, temperature, humidity, and operating time. If necessary, the system can be modified by changing its speed, stopping it, or restarting it. With this option, the system can be remotely stopped by an authorized person before any intervention is made, or restarted at the desired speed. In the present study, the rotational speed was set to 5 RPM at a motor supply voltage of 12 V prior to culture initiation and maintained constant throughout the 14-day culture period, consistent with the approach reported in comparable rotating bioreactor studies [16]. This speed was selected to generate low-shear laminar flow conditions while ensuring adequate nutrient convection throughout the construct. The monitored parameters served to verify stable system operation rather than to guide real-time adjustments; parameter modification would only be required in the event of a system malfunction. Furthermore, the device is equipped with sensors that trigger visual and auditory alerts in the event of motor failure. Specifically, a magnetic Hall effect sensor continuously monitors the rotational pulse frequency. If the expected pulse rate is not detected because of motor stall, mechanical jam, or unexpected power interruption, the system automatically activates onboard auditory and visual alarms and simultaneously sends a push notification to the operator’s mobile device, enabling prompt corrective intervention without opening the incubator. The modular cell culture column consists of eight distinct parts, following the structural model previously described by Meghezi et al. [30].

### 2.2. Production and Assembly of the Bioreactor

The production phase initiated with a preliminary prototype, which was essential for verifying the mechanical alignment and overall functionality of the assembly (Figure 2). We refined the holder and catcher components through several testing iterations to achieve a more reliable grip and precise axial positioning. Our study used Polylactic Acid (PLA) material as a preliminary prototype. However, PLA was chosen for the preliminary model trial because it does not cause problems at these low temperatures and humidity conditions and is non-toxic. Furthermore, in this method, there is no direct interaction between the samples and cell populations and the materials produced by FDM printing. Additionally, in the final studies, safer conditions were achieved by using a more biocompatible Polyethylene Terephthalate Glycol (PETG) type filament. PETG offers superior resistance to hydrolysis and higher dimensional stability under warm and humid incubator conditions compared to PLA, making it more suitable for prolonged culture periods. For the final structural frame, we utilized PETG as the primary material, processed via an Ender 3 Pro 3D printer (Creality, Shenzhen, China).

To drive the mechanical rotation, a JGY-370 geared DC motor was selected, providing a torque of 14 kg·cm at 12 V. The control logic is handled by an ESP-12E microcontroller (ESP8266-based, Ai-Thinker, Shenzhen, China), which communicates with a STMicroelectronics L293D full-bridge motor driver (STMicroelectronics, Geneva, Switzerlan). These electronic modules were integrated onto a dedicated ESP12E motor shield to simplify the wiring and ensure stable connections. Although this specific study utilized a single-channel setup, the system’s modular architecture can support 1.2 A, allowing for future expansions where multiple reactors could run with independent speed profiles.

We secured the system’s operational reliability by integrating a magnetic Hall effect sensor. This component monitors the rotation in real-time and acts as a diagnostic tool for detecting mechanical inconsistencies. In the event of a malfunction, including motor stall, mechanical jam, or unexpected power interruption, the system is programmed to automatically activate onboard auditory and visual alarms and simultaneously transmit a mobile notification to the operator. Furthermore, the IoT-enabled control unit permits remote management through an HTML-based data transfer protocol. This allows users to oversee rotational parameters via a smartphone, a feature that we found crucial for maintaining a sterile environment by minimizing incubator openings. Additionally, the HMI can be positioned outside the incubator, ensuring that monitoring remains accessible without compromising the internal culture conditions.

### 2.3. Sterilization of the Bioreactor

The bioreactor components were sterilized using ethylene oxide (EtO) gas at the Dokuz Eylül University Research and Application Hospital. EtO was selected for its high efficacy in penetrating the complex geometries and 3D-printed materials of the assembly, ensuring comprehensive decontamination. This cold sterilization method is particularly advantageous for the heat-sensitive polymer components used in this study, as it achieves high-level sterility without compromising the structural or mechanical integrity of the bioreactor.

### 2.4. Fabrication of the 3D Artificial Blood Vessel Model

The outer framework of the 3D vascular tissue model was engineered using two distinct layered formulations to ensure structural integrity and biomimetic properties. Initially, 2% (*w*/*v*) agarose was dissolved in 0.1% sodium hydroxide (NaOH) to provide the necessary mechanical stability. A hybrid scaffold structure was then developed by blending 3% (*w*/*v*) chitosan and 4% (*w*/*v*) agarose solutions at a 1:1 (*v*/*v*) ratio. These prepared mixtures were cast into tubular molds within a sterile laminar flow cabinet and allowed to polymerize at room temperature to fix the tubular geometry.

Commercial HASMC (ATCC, Manassas, VA, USA) were employed as the cellular component, cultured in a humidified incubator at 37 °C with 5% CO_2_ according to the manufacturer’s protocols. HASMCs were selected as the primary cell type because smooth muscle cells constitute the predominant cellular component of the tunica media of native blood vessels and are directly involved in vascular wall mechanics, contractility, and remodeling. For an initial proof-of-concept validation of a new bioreactor platform, HASMC viability and metabolic activity provide meaningful and interpretable biological endpoints. Upon reaching approximately 80% confluence, the cells were harvested using enzymatic methods and resuspended in fresh culture media to achieve a target seeding density of 2 × 10^6^ cells/mL. This cell suspension was directly seeded onto the lumen and external surfaces of the freshly polymerized scaffolds. Following cell seeding, the constructs were maintained under static conditions for 24 h to allow initial cell attachment to the scaffold surface, consistent with the standard adhesion period recommended for HASMC cultures [ATCC]. To evaluate the impact of mechanical stimulation on functional vascular development, the seeded scaffolds were divided into two experimental groups: a dynamic group, mounted in a rotating bioreactor for 14 days under constant rotation and continuous media exchange, and a static control group maintained in standard tissue culture vessels. Culture medium was exchanged every 48 h by withdrawing spent medium and replenishing with fresh pre-warmed medium via syringe through the dedicated medium exchange port, consistent with standard protocols reported for rotating bioreactor systems [18].

### 2.5. Biocompatibility Assessment and Cytotoxicity Analysis of the Bioreactor Environment

To evaluate the potential cytotoxic effects of the agarose-chitosan-based scaffold and the mechanical rotation within the bioreactor, an LDH leakage assay was performed. Given that LDH is a cytosolic enzyme released only upon cell membrane compromise, its concentration in the culture medium serves as a reliable indicator of cell death. Supernatant samples were collected from both the dynamic (bioreactor) and static (control) groups on Days 1, 7, and 14 to longitudinally monitor the system’s biocompatibility. LDH activity was quantified using a commercial kit (Sigma-Aldrich, St. Louis, MO, USA) in accordance with the manufacturer’s protocol. Briefly, the collected media were reacted with the LDH reagent, and the absorbance was measured at 490 nm using a microplate reader. To ensure accurate quantification, a Negative Control (NC) representing spontaneous LDH release and a Positive Control (PC) representing maximum LDH release following total cell lysis were established for both experimental conditions. The percentage of cytotoxicity and subsequent cell viability was calculated using the following equations:

Cytotoxicity (%) = [(Net Sample − Net NC)/(Net PC − Net NC)] × 100

Cell Viability (%) = 100 − Cytotoxicity

Background absorbance from cell-free hydrogel constructs was subtracted from all readings to obtain the “net” values used in the formulas. All experiments were performed using five independent samples (n = 5), and the data are presented as mean ± standard deviation.

### 2.6. Confocal Imaging of the 3D Vascular Structure

To evaluate the cellular architecture within the 3D vascular tissue, specimens were precisely sectioned using a biopsy punch. Cellular visualization was achieved by staining actin filaments with MitoTracker Deep Red (MTDR; Molecular Probes, Invitrogen, Carlsbad, CA, USA) according to the manufacturer’s protocols. The HASMC-seeded hydrogel constructs were incubated with MTDR for 30 min at 37 °C. Following incubation, the scaffolds were washed twice with phosphate-buffered saline (PBS) at room temperature to remove residual dye.

To preserve cellular morphology and maintain structural stability, the samples were fixed in freshly prepared 4% paraformaldehyde (PFA) for 15 min in a dark environment. Post-fixation, the constructs were washed again with PBS to eliminate any remaining fixative. Finally, the samples were mounted using a medium containing DAPI (4′,6-diamidino-2-phenylindole; UltraCruz^®^, Santa Cruz Biotechnology, Inc., Dallas, TX, USA) to label the cell nuclei.

High-resolution images of the cell distribution and structure were obtained using a Zeiss LSM880 confocal microscope (Carl Zeiss AG, Oberkochen, Germany). Z-stack images were acquired, and the slice thickness was optimized using ZEN software (ZEN 3.10, Carl Zeiss AG, Oberkochen, Germany). Background autofluorescence from the agarose-chitosan matrix was reduced during ImageJ (NIMH, Bethesda, MD, USA) analysis. The images were further processed and analyzed using ZEN software to improve image quality and perform quantitative analysis.

## 3. Results

### 3.1. Manufacture and Assembly of the Bioreactor

**Figure 4:**
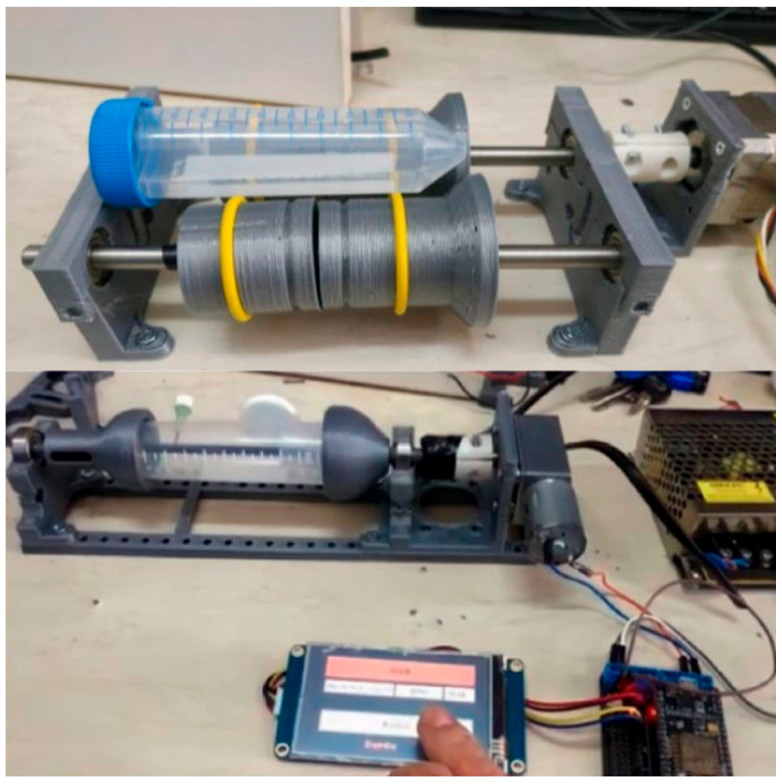
Fabricated bioreactor prototype produced by 3D printing and integrated with electronic components for preliminary system testing.

All components of the designed bioreactor were modeled using the 123Design software (Autodesk) and fabricated using three-dimensional printing. Following fabrication, the printed parts were integrated with the electronic components to enable preliminary system testing (Figure 4).

In the initial design phase, the tissue culture tube was implemented without fixed connectors, and a tumbler-based rotation approach was evaluated. However, during preliminary testing, the presence of tube connectors was found to interfere with stable rotation. To overcome this limitation, the design was revised to incorporate a two-sided holder configuration, which provided improved mechanical stability during rotation.

In addition, a servo motor was initially used to drive the rotating system. Due to its discrete motion, this configuration introduced unwanted vibration that was not suitable for continuous low-speed rotation. Therefore, the servo motor was replaced with a geared DC motor, allowing smooth and continuous axial rotation of the sample holder. This final motor configuration is shown in Figure 4.

**Figure 5:**
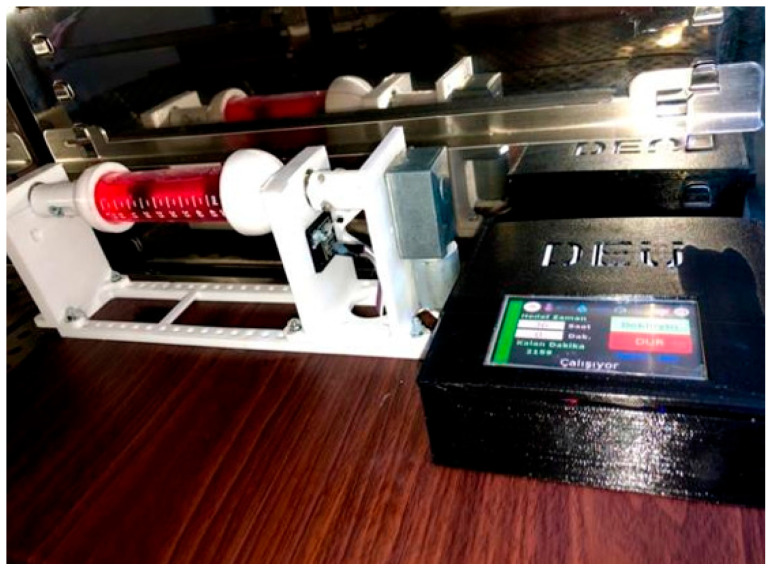
Bioreactor system operating under incubator conditions.

To improve flexibility for different experimental setups, the height of the tissue culture tube was increased in the final design to accommodate larger or alternative constructs. The finalized design and its implementation are presented in Figure 5.

**Figure 6:**
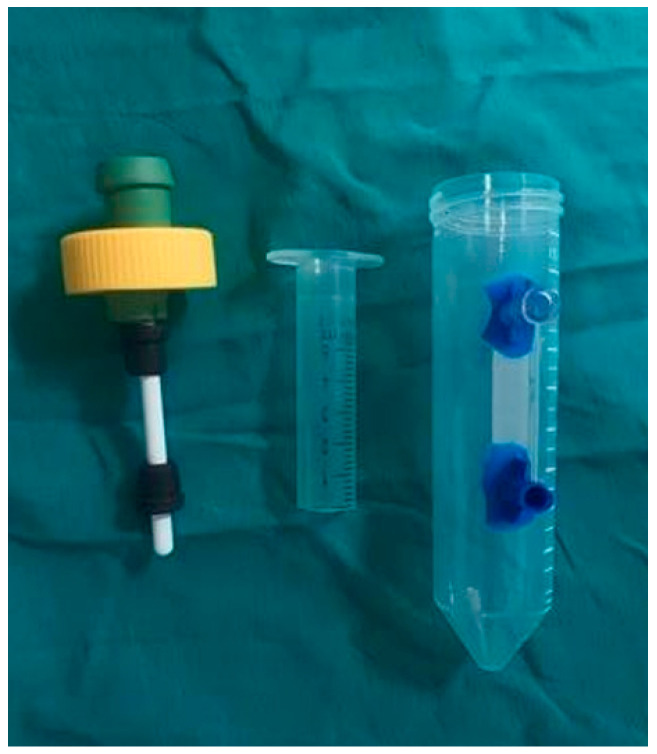
Fabricated a 3D cell culture chamber corresponding to the schematic design presented in Figure 2.

The sample holder was manufactured according to the finalized design specifications (Figure 6). The mandrel diameter was 4.5 mm with a total length of 100 mm, while the remaining section containing the stopper components measured 37 mm in length. To ensure leak-free operation during long-term experiments, the holder assembly was sealed using medical-grade silicone and tested prior to use.

**Figure 7:**
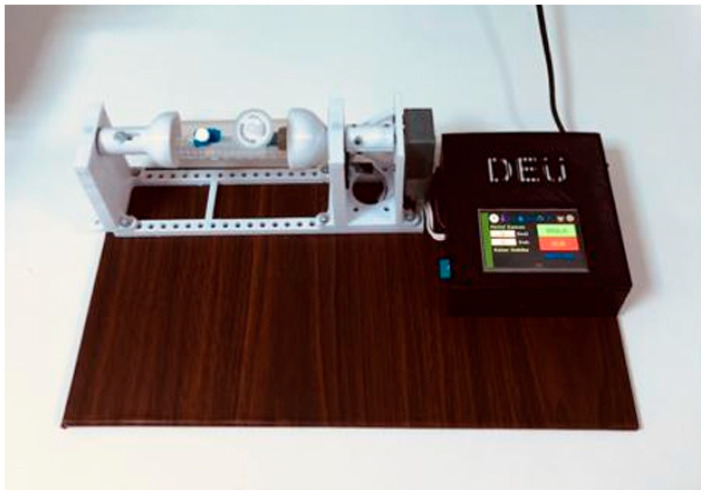
Fully assembled bioreactor system with integrated human–machine interface (HMI) for system control and parameter adjustment.

Following sterilization, all components were assembled to form the complete bioreactor system. The fully assembled bioreactor and the associated human–machine interface (HMI) control screen are shown in Figure 7.

### 3.2. Three-Dimensional Artificial Blood Vessel Formation and Culture in the Rotating Bioreactor

To create the framework for our vascular model, we prepared a 2% (*w*/*v*) agarose solution under sterile conditions. Since agarose solidifies quickly at room temperature, we used a heated magnetic stirrer in the laminar flow cabinet during the process to keep the solution liquid. Then, we carefully poured the liquid agarose around a cylindrical mandrel and let it sit for 10 min to form a stable tubular hydrogel structure. In parallel, we collected HASMCs when they reached 85% confluence. After centrifugation, we resuspended the cell pellet in fresh culture medium and placed it on the surface of a pre-prepared agarose tube. We allowed the cells to settle and adhere to the scaffold for a short period before transitioning to the dynamic culture phase. After the cells were added, the holder was placed into the bioreactor, and the entire assembly was placed in a humid incubator (37 °C, 5% CO_2_). Initially, we kept the system static to ensure cellular adhesion, and then we switched to continuous rotation mode (Figure 7).

Our bioreactor design uses a cylindrical chamber, completely filled with culture medium, where the structure slowly rotates around its horizontal axis. This special spinning arrangement is crucial; it creates a low shear, low turbulence environment that mimics physiological conditions while preventing cellular collapse. Rotating wall vessel bioreactor systems are known to operate at low Reynolds numbers, generating laminar flow conditions that support cell attachment and viability [16]. We found that this gentle motion significantly improved nutrient exchange and mass transport by ensuring that the cells were uniformly exposed to the environment without the stress of high-speed turbulence. An externally mounted motor provided the rotational motion; this allowed us to precisely control the speed throughout the maturation period (Figure 5).

### 3.3. Confocal Analysis of Cellular Distribution and Mitochondrial Activity

**Figure 8:**
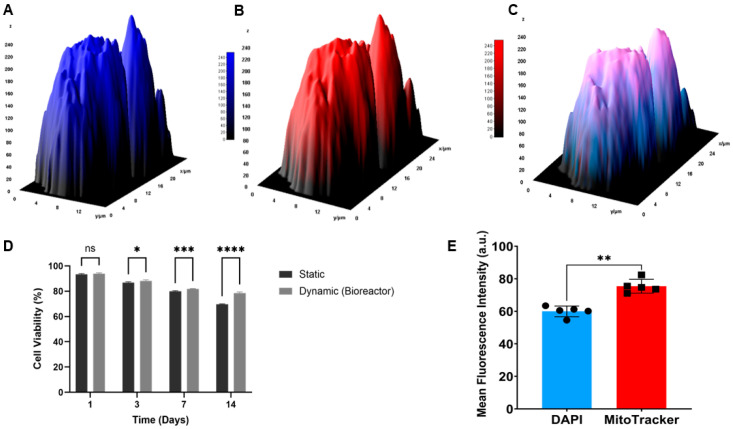
Representative confocal micrograph (merged DAPI + MitoTracker channels) of HASMC-seeded constructs under dynamic conditions at Day 7. (**A**) DAPI Three-dimensional surface plot. (**B**) MitoTracker channel showing mitochondrial activity. (**C**) Merged channel 3D surface plot showing co-localization of signals. (**D**) Cell viability (%) under static and dynamic conditions over 14 days; mean ± SD (n = 5); two-way ANOVA with Šídák’s test (* *p* < 0.05, *** *p* < 0.001, **** *p* < 0.0001; ns: not significant). (**E**) Mean fluorescence intensity of DAPI vs. MitoTracker at Day 7; mean ± SD (n = 5); unpaired *t*-test (** *p* < 0.01). 3D plot intensities normalized for qualitative visualization.

The agarose-chitosan tubular scaffolds used in this study had an inner diameter of 4.5 mm, determined by the mandrel geometry, and a functional length of 78 mm. Confocal microscopy demonstrated a widespread distribution of cells throughout the 3D hydrogel constructs under dynamic culture conditions. DAPI staining enabled clear visualization of cell nuclei (Figure 8A), while MitoTracker staining indicated active mitochondrial function within the constructs (Figure 8B). The merged fluorescence image (Figure 8C) confirms the spatial co-distribution of nuclear and mitochondrial signals within the hydrogel matrix. The acquired images were analyzed using ImageJ software (NIH, Bethesda, MD, USA), and fluorescence intensity values for each channel were quantified. To further visualize spatial distribution, 3D surface plots were generated using the Interactive 3D Surface Plot plugin (Figure 8A–C), providing a qualitative representation of fluorescence intensity across the constructs.

The 3D surface analysis revealed a higher fluorescence intensity in the MitoTracker channel compared to DAPI, suggesting increased mitochondrial activity in dynamically cultured cells. This observation was supported by quantitative analysis, which showed a statistically significant increase in MitoTracker fluorescence intensity compared to DAPI (*p* < 0.01) (Figure 8E).

Overall, these findings indicate that dynamic bioreactor culture not only preserves cell viability but also enhances cellular metabolic activity within the 3D hydrogel structures. These results are consistent with the observed high cell viability under dynamic conditions.

The LDH test was conducted to quantitatively assess the integrity of the cell membrane under dynamic culture conditions over a 14-day period. The results support that mechanical rotation in the bioreactor did not induce significant cytotoxic effects.

On the first day, a baseline cytotoxicity of 8.6 ± 1.2% was observed due to initial cell seeding and processing procedures. Later, as the cells adapt to the dynamic environment, the levels of cytotoxicity gradually decrease. On the 14th day, cytotoxicity decreased to 4.2 ± 0.5%, confirming that the low shear conditions of the bioreactor supported long-term cell viability. These findings indicate that the dynamic culture system maintains biocompatibility and preserves cell integrity (Figure 8D).

## 4. Discussion

The findings of this study demonstrate that the developed rotating bioreactor system provides a biologically supportive and functionally stable microenvironment for 3D vascular tissue engineering applications. The LDH assay results revealed a progressive decrease in cytotoxicity over time, while cell viability remained above 90% throughout the 14-day culture period, indicating that the dynamic culture conditions preserved cell membrane integrity and supported cellular adaptation. These findings are consistent with previous reports showing that dynamic bioreactor culture supports cell membrane integrity and cellular adaptation under mechanical stimulation [11,30]. The low-shear rotational environment generated by the present system may also contribute to stable smooth muscle cell attachment, as excessive shear stress has been associated with impaired cell adhesion in rotating bioreactor systems [16].

Confocal fluorescence imaging confirmed the presence of HASMC within the hydrogel constructs under dynamic culture conditions, as evidenced by DAPI-stained nuclei and MitoTracker-labeled mitochondria distributed throughout the scaffold matrix. Quantitative fluorescence analysis showed significantly higher MitoTracker intensity compared to DAPI (*p* < 0.01), indicating enhanced mitochondrial activity in dynamically cultured cells. This suggests that the applied mechanical stimulation not only preserved cell viability but also promoted cellular metabolic activity. Similar findings have been described in previous rotating bioreactor studies, where dynamic culture conditions promoted cell proliferation and reduced apoptosis compared to static culture [16,31]. In addition, the 3D surface plot analysis provided spatial visualization of fluorescence distribution throughout the construct, supporting the presence of retained cells within the hydrogel matrix. Nevertheless, confocal imaging was limited to the dynamic culture group and a single experimental time point, representing a limitation of the present study.

Bioreactor systems used in vascular tissue engineering are commonly based on perfusion-driven designs, which improve nutrient transport through direct flow. However, these systems are often associated with high cost, complex setup, and demanding maintenance requirements [4]. In contrast, the rotating bioreactor system developed in this study offers a simpler and more cost-effective alternative while still providing improved mass transfer through continuous motion. Previous comparative studies have suggested that rotating bioreactors may improve extracellular matrix deposition and cell distribution in tubular vascular constructs compared to perfusion-based systems [30]. In the present design, the fully filled culture chamber eliminates the air-liquid interface and maintains continuous contact between the construct and the culture medium throughout rotation.

A key innovation of the present system is the integration of a Wi-Fi-supported control mechanism and a touchscreen-based human–machine interface (HMI), enabling real-time monitoring and remote parameter adjustment. Many existing bioreactor systems remain limited in terms of remote accessibility and real-time control [28,32]. The ability to control the system without opening the incubator reduces contamination risk and improves experimental stability, particularly during long-term culture. Recent studies have emphasized the increasing use of IoT-assisted monitoring strategies in bioreactor systems to improve process stability and reduce operator-related variability [32,33]. The use of FDM-based fabrication further supports the development of accessible and customizable bioreactor platforms that can be rapidly adapted for specific experimental applications [20,34].

In addition, recent studies focusing on vascular bio-fabrication and engineered vessel maturation further support the increasing relevance of modular and adaptable bioreactor systems for translational vascular tissue engineering applications [32,33,34].

HASMCs were selected because smooth muscle cells represent the predominant cellular component of the tunica media and play a central role in vascular wall function and remodeling [4]. As an initial proof-of-concept study, the present work focused on evaluating the ability of the bioreactor system to maintain cell viability and metabolic activity under dynamic culture conditions rather than generating a fully mature vascular graft. Future investigations will incorporate endothelial co-culture models and vascular maturation markers to further evaluate tissue development within the system.

Despite these promising findings, several limitations should be acknowledged. Confocal imaging was performed only in the dynamic culture group, and comparative multi-depth analysis with static controls was not included. In addition, key biophysical parameters such as shear stress and oxygen gradients were not directly quantified. Mechanical characterization, extracellular matrix analysis, and smooth muscle phenotypic marker expression were also beyond the scope of the present proof-of-concept study. Furthermore, biological validation was limited to a 14-day culture period. Future investigations will incorporate endothelial co-culture models and vascular maturation markers to further evaluate tissue development within the system, consistent with recent advances in functional 3D-bioprinted vascular constructs [26].

The static culture condition used in this study served as a baseline control for the initial validation of the bioreactor system. Future studies incorporating additional dynamic culture platforms may provide a broader comparison of system performance.

Overall, the findings suggest that the developed rotating bioreactor system represents an accessible and adaptable platform for 3D vascular tissue engineering applications, combining low-cost fabrication, remote monitoring capability, and biologically supportive dynamic culture conditions.

## 5. Conclusions

In this study, a modular and cost-effective rotating bioreactor system equipped with a Wi-Fi-supported control unit and a touchscreen human–machine interface (HMI) was successfully developed and biologically validated for 3D vascular tissue engineering applications. The results demonstrated that dynamic culture conditions preserved cell viability, promoted widespread cellular distribution, and enhanced mitochondrial activity within 3D hydrogel constructs. Compared to conventional perfusion-based systems, the proposed platform offers a simpler and more accessible alternative while maintaining effective mass transfer through continuous low-speed rotation. The integration of remote monitoring and control further improves experimental stability and reduces contamination risks during long-term culture.

Overall, the developed system provides a practical and scalable approach for in vitro vascular modeling and represents a promising platform for future tissue engineering and translational research applications. Future investigations will focus on further biological and biomechanical characterization to evaluate the long-term potential of the system for vascular tissue engineering applications.

## Data Availability

The original contributions presented in the study are included in the article, further inquiries can be directed to the corresponding author.
